# Chest CT opportunistic biomarkers for phenotyping high-risk COVID-19 patients: a retrospective multicentre study

**DOI:** 10.1007/s00330-023-09702-0

**Published:** 2023-05-11

**Authors:** Anna Palmisano, Chiara Gnasso, Alberto Cereda, Davide Vignale, Riccardo Leone, Valeria Nicoletti, Simone Barbieri, Marco Toselli, Francesco Giannini, Marco Loffi, Gianluigi Patelli, Alberto Monello, Gianmarco Iannopollo, Davide Ippolito, Elisabetta Maria Mancini, Gianluca Pontone, Luigi Vignali, Elisa Scarnecchia, Mario Iannaccone, Lucio Baffoni, Massimiliano Spernadio, Caterina Chiara de Carlini, Sandro Sironi, Claudio Rapezzi, Antonio Esposito

**Affiliations:** 1 Experimental Imaging Center, IRCCS San Raffaele Scientific Institute, Via Olgettina 60, Milan, Italy; 2 School of Medicine, Vita-Salute San Raffaele University, Via Olgettina 58, 20132 Milan, Italy; 3 GVM Care & Research Maria Cecilia Hospital, Cotignola, Italy; 4 Ospedale Di Cremona, Cremona, Italy; 5 ASST Bolognini Hospital, Bergamo Est, Italy; 6 Guglielmo da Saliceto Hospital, Piacenza, Italy; 7 Ospedale Maggiore, Bologna, Italy; 8 San Gerardo Hospital, Monza, Italy; 9 Centro Cardiologico Monzino IRCCS, Milan, Italy; 10 Parma University Hospital, Parma, Italy; 11 ASST Valtellina and Alto Lario, Eugenio Morelli Hospital, Sondalo, Italy; 12 San Giovanni Bosco Hospital, ASL Città Di Torino, Turin, Italy; 13 Casa Di Cura Villa Dei Pini, Civitanova Marche, Italy; 14 ICC Istituto Clinico Casalpalocco, Rome, Italy; 15 San L. Mandic Hospital, Merate, Italy; 16 ASST Papa Giovanni XXIII, Bergamo, Italy; 17 Azienda Ospedaliero-Universitaria Di Ferrara, Cona, FE Italy

**Keywords:** COVID-19, Sarcopenia, Fatty liver, Coronary artery disease, Computed tomography

## Abstract

**Objective:**

To assess the value of opportunistic biomarkers derived from chest CT performed at hospital admission of COVID-19 patients for the phenotypization of high-risk patients.

**Methods:**

In this multicentre retrospective study, 1845 consecutive COVID-19 patients with chest CT performed within 72 h from hospital admission were analysed. Clinical and outcome data were collected by each center 30 and 80 days after hospital admission. Patients with unknown outcomes were excluded. Chest CT was analysed in a single core lab and behind pneumonia CT scores were extracted opportunistic data about atherosclerotic profile (calcium score according to Agatston method), liver steatosis (≤ 40 HU), myosteatosis (paraspinal muscle F < 31.3 HU, M < 37.5 HU), and osteoporosis (D12 bone attenuation < 134 HU). Differences according to treatment and outcome were assessed with ANOVA. Prediction models were obtained using multivariate binary logistic regression and their AUCs were compared with the DeLong test.

**Results:**

The final cohort included 1669 patients (age 67.5 [58.5–77.4] yo) mainly men 1105/1669, 66.2%) and with reduced oxygen saturation (92% [88–95%]). Pneumonia severity, high Agatston score, myosteatosis, liver steatosis, and osteoporosis derived from CT were more prevalent in patients with more aggressive treatment, access to ICU, and in-hospital death (always *p* < 0.05). A multivariable model including clinical and CT variables improved the capability to predict non-critical pneumonia compared to a model including only clinical variables (AUC 0.801 vs 0.789; *p* = 0.0198) to predict patient death (AUC 0.815 vs 0.800; *p* = 0.001).

**Conclusion:**

Opportunistic biomarkers derived from chest CT can improve the characterization of COVID-19 high-risk patients.

**Clinical relevance statement:**

In COVID-19 patients, opportunistic biomarkers of cardiometabolic risk extracted from chest CT improve patient risk stratification.

**Key Points:**

**•** *In COVID-19 patients, several information about patient comorbidities can be quantitatively extracted from chest CT, resulting associated with the severity of oxygen treatment, access to ICU, and death.*

**•** *A prediction model based on multiparametric opportunistic biomarkers derived from chest CT resulted superior to a model including only clinical variables in a large cohort of 1669 patients suffering from SARS- CoV2 infection.*

**•** *Opportunistic biomarkers of cardiometabolic comorbidities derived from chest CT may improve COVID-19 patients’ risk stratification also in absence of detailed clinical data and laboratory tests identifying subclinical and previously unknown conditions.*

## Introduction

During the COVID-19 outbreak, chest CT was widely used for its excellent sensitivity in diagnosing SARS-CoV2-associated pneumonia [1], resulting in becoming particularly useful to speed up the diagnosis in overwhelmed emergency departments (ED) and in community transmission scenarios [1, 2].

In comparison to chest X-ray, chest CT has the advantage to provide a differential diagnosis in symptomatic patients [1] and to identify acute complications of COVID-19 infection, especially pulmonary embolism [3].

Chest CT was also able to provide prognostic information; in fact, pneumonia extension and its attenuation features, especially semi-consolidation and consolidation, have been associated with disease severity, oxygen exchange impairment, and patient outcome [4, 5]. Therefore, in order to improve patients’ risk stratification, several prognostic models combined chest CT biomarkers of COVID-19 pneumonia severity together with clinical predictors of COVID-19 outcome, such as age, sex, comorbidities, and laboratory biomarkers of systemic inflammation and multiple organ damage [6]. Despite their potential utility, these prediction scores were hardly applicable in clinical routine due to methodological flaws [6], including the number of variables needed (leading to potentially huge numbers of missing variables), the long turnaround time of required lab tests, and the lack of reference values standardization among laboratories, and the often challenging collection of patients past medical history and comorbidities in the overwhelmed EDs. However, knowledge about pre-existing chronic conditions would be important, since they severely affect the clinical course of COVID-19 and potentially its post-acute sequelae [7-10].

Chest CT could potentially overcome these limitations, providing additional opportunistic information about patients’ cardiovascular and metabolic comorbidities [10-12] as well as about patient fragility [13], thanks to the direct and objective assessment of organ attenuation modifications. In fact, the assessment of tissue Hounsfield units (HU) is able to identify modifications of tissue composition. In particular, CT allows detection with high sensitivity and tissue alterations related to calcium and fat content due to the significant difference in attenuation values of fat, calcium, and soft tissue. CT can quantify the accumulation of calcium in coronary arteries, a known marker of atherosclerosis [14] and vascular senescence that can be easily derived from non-ECG gated chest CT, which resulted associated with an increased risk of cardiovascular events and all-cause mortality [15]. Furthermore, CT can also detect the reduction in bone calcium content: in fact, the reduction in trabecular CT attenuation of vertebral bones on routine chest and/or abdomen CT examinations has been associated with osteoporosis [16]. Similarly, soft tissue fatty infiltration can be easily measured with CT, allowing to identify myosteatosis, a marker of muscle low quality [17] associated with respiratory functional impairment [18] and dysmetabolism [19] and liver steatosis, which has been associated with an abnormal lipid profile, abnormal liver function, and certain comorbidities such as diabetes mellitus and alcohol consumption [20].

Hence, considering the wide spectrum of information that could be potentially extracted from routine chest CT, the aim of the present study was to investigate the incremental value of a multiparametric CT analysis over clinical data for the phenotypization of high-risk patients aimed to improve conventional method of risk stratification of patients affected by COVID-19.

## Methods

### Study design and participants

This is a multicenter retrospective cohort study.

Consecutive adult patients (age 18 years or older) with RT-PCR-confirmed SARS-CoV-2 infection submitted to chest CT within 72 h from admission in fifteen tertiary-level hospitals located in Middle and Northern Italy between March 1^st^ and April 20^th^, 2020.

Exclusion criteria were contrast-enhanced CT scan and missing outcome data at 30-day follow-up.

The study was approved by the local ethics committees and written informed consent was obtained.

Each centre provided patients’ clinical data by filling out a centralized electronic case report form and sent chest CT images to San Raffaele Hospital for centralized image analysis.

Collected clinical data were demographic parameters (age and sex), comorbidities (hypertension, diabetes, chronic lung disease, cardiovascular disease), laboratory tests (white blood cell count “WBC”, creatinine, C-reactive protein “CRP,” lactate dehydrogenase “LDH,” troponin I, interleukin-6, and D-dimer) and outcome data (need for oxygen therapy, need for orotracheal intubation, and death).

### Chest CT scan

All chest CT examinations were performed on multidetector scanners with at least 16 detector rows. All volumetric chest scans were reformatted at a 2.5-mm slice thickness without gap or overlap. Images were reconstructed with a sharp kernel for lung parenchyma evaluation and with a soft kernel for mediastinum evaluation, and they were visualized using standard windows (lung: width 1400 HU, center − 450 HU; mediastinum: width 350 HU, center 40 HU).

Chest CT analysis was performed by a radiologist with 10 years of experience in cardiothoracic imaging, blinded to clinical data, using dedicated software (IntelliSpace Portal 8.0, Philips Healthcare).

The following parameters were extracted:

- Parameters of lung involvement:
  • Pneumonia extension score from 0 to 4 (0% lung involvement: absent, score 0; 1–25%: minimal, score 1; 26–50%: mild, score 2; 51–75%: moderate, score 3; and > 75%: severe, score 4) [21].
- Parameters of cardiovascular risk:
  • Coronary artery calcium score according to Agatston, automatically quantified by detecting adjacent pixels with an area ≥ 1 mm^2^ and a density above 130 HU [8, 12, 21, 22]. Patients were classified into three classes (low-risk: 0–10; intermediate-risk: 10–1000; high-risk: ≥ 1000). Patients with evidence of coronary stents and coronary artery bypass grafting (CABG) were classified in the high-risk class together with patients with an Agatston score ≥ 1000.
- Parameters of metabolic alteration and fragility:
  • Liver moderate-to-severe steatosis, defined as a mean liver attenuation ≤ 40 HU [23] manually measured by drawing two regions of interest (ROIs) on the right and left lobes, excluding vessels.
  • Myosteatosis, defined as a sex-specific mean muscle attenuation < 31.3 HU for females and < 37.5 HU for males, manually measured drawing the cross-sectional areas of the paravertebral muscles on both sides of the spine at the D12 level, considering erector spinae, longissimus thoracis, spinalis thoracis, and iliocostalis lumborum muscles [24].
  • Osteoporosis, defined as a mean bone attenuation at D12 level < 135 HU, as previously reported [16, 25], was manually measured by drawing an ROI on D12 vertebral bone for trabecular attenuation measurement. If the D12 level was unsuitable for measurement (e.g., because of a compression fracture), the measurement was conducted at the D11 level.

### Statistical analysis

Categorical variables were reported as numbers and percentages, continuous variables as mean and standard deviation or median and interquartile range (IQR) according to their distribution, assessed with the Kolmogorov–Smirnov test. ANOVA, chi-squared or the Mann–Whitney U tests were used to compare variables’ distribution between groups for categorical or continuous variables, respectively. Follow-up data were censored 30 days from hospital admission. Overall survival according to critical illness (pneumonia extent ≤ 50% vs pneumonia extent > 50%) was estimated with the Kaplan–Meier curves and groups were compared with the log-rank test. To identify the potential power of collected variables predicting the dichotomous outcome (in-hospital death vs hospital discharge), we used a multivariate binary logistic regression with the “backward elimination” method. Results are presented as adjusted odds ratios (ORs) and 95% confidence intervals (95% CIs). Variables were tested for collinearity, and the variance inflation factor was assessed. The Hosmer and Lemeshow test was performed, and R2 values were evaluated with Cox and Snell and Nagelkerke methods for each regression analysis. No data imputation for missing values was used. The performance of the obtained models to predict the outcome was assessed using receiver operating characteristic (ROC) curve analysis; the area under the ROC curves (AUCs) was compared with the DeLong method.

A *p* value < 0.05 was considered statistically significant; when multiple testing was performed, Bonferroni correction was applied.

Analyses were performed using SPSS v.26.0 (IBM SPSS Inc.).

## Results

### Population characteristics

A total of 1845 consecutive COVID-19 patients fulfilled the inclusion criteria. Of 1845 patients, 176 were excluded for missing outcomes. Hence, the final cohort included 1669 patients.

Demographic and clinical characteristics of the overall population are detailed in Table 1 and, according to outcome, in Table 2.

**Table 1:** Demographic, comorbidities, and imaging characteristics

Demographics			
Male sex (*n*, %)			1105/1669 (66.2%)
Age (mean ± SD)			67.52 ± 13.2
**Laboratory tests**			
WBC value at admission (WBC/mm^3^)			6800 [4985–9850]
Hb value at admission (g/dL)			13.7 [12.3–14.8]
LDH value at admission (U/L)			349 [255–470]
CRP value at admission (mg/dL)			9.0 [3.2–18]
SatO_2_ at admission (%)			92 [88–95]
D-dimer peak value (µg/L)			4 [4–18.5]
TnI peak value (ng/L)			52 [10–185]
**Comorbidities (%)**			
Presence of any comorbidity (*n*, %)			1150/1669 (68.9%)
Cardiovascular diseases (*n*, %)	Hypertension		937/1658 (56.5%)
	Heart diseases		228/1633 (14%)
	Previous PTCA		127/1669 (7.6%)
	Previous CABG		50/1250 (4%)
	Peripheral artery disease		102/1657 (6.2%)
Diabetes (*n*, %)			310/1658 (18.7%)
Chronic obstructive pulmonary disease (*n*, %)			174/1657 (10.5%)
Oncological history (*n*, %)			74/1656 (4.5%)
Chronic renal failure^ (*n*, %)			99/1238 (8%)
**CT findings**			
**Lung**	Pneumonia score*		2 [1–3]
	Pneumonia score > 50%		532/1669 (31.9%)
**Cardiovascular**	Agatston score		24.4 [0–261.7]
	Agatston score 0 < 10		675/1542 (43.8%)
	Agatston score ≥ 10 < 1000		729/1542 (47.3%)
	Agatston score ≥ 1000		138/1542 (8.9%)
	Agatston score ≥ 1000, PCI and/or CABG		265/1669 (15.9%)
**Skeletal muscle**	Median HU_D12_		40.3 [31.2–47.3]
	Patients with sarcopenia**		582/1661 (35%)
**Liver parenchyma**	Median liver HU		47.4 [38.1–53.6]
	Patients with hepatic steatosis***		465/1664 (27.9%)
**Lumbar spine bone**	Median bone density HU_D12_		127.0 [94.1–164.2]
	Patients with osteoporosis****		910/1642 (55.4%)
**Hospital stay and outcome**			
Non hospitalized patients (*n*, %)			73/1669 (4.4%)
Hospitalization length (days)			14 [8–23]
Oxygen therapy (*n*, %)		Admitted without oxygen therapy	161/1669 (9.6%)
		Admitted with oxygen therapy	543/1669 (32.5%)
		Admitted with non-invasive ventilation	269/1669 (16.1%)
		Admitted with the need for invasive ventilation	199/1669 (11.9%)
Patients admitted to intensive care unit (*n*, %)			199/1669 (11.9%)
Deceased Patients (n, %)			424/1669 (25.4%)

^ eGFR < 60

^*^Semiquantitative evaluation from 0 to 4, as follows: 0, 0% extension; 1, 1–25% extension; 2, 26–50% extension; 3, 51–75% extension; 4, over 75% extension

^**^cut-off value of 31.3 HU in Females and 37.5 HU in Males

^***^ cut-off value 40 HU

^****^ cut-off value of 134 HU

Data are reported as median [Interquartile range] except otherwise specified

**Table 2:** Demographic characteristics and comorbidities in discharged and deceased patients

	Discharged (*n* = 1245)	Deceased (*n* = 424)	*p* value	Adj. *p *value
Male sex	789/1245 (63.4%)	315/424 (74.3%)	< 0.0001	** < 0.0001**
Age	64.8 ± 13.1	75.4 ± 10.2	< 0.0001	** < 0.0001**
WBC value at admission, WBC/mm^3^	6590 [4900–9400]	7350 [5420–10910]	< 0.0001	** < 0.0001**
Hb value at admission, g/dL	13.8 [12.4–14.9]	13.3 [12–14.5]	0.001	**0.019**
LDH value at admission, U/L	328 [244.8–427.3]	450 [312.5–601]	< 0.0001	** < 0.0001**
CRP value at admission, mg/dL	7.6 [2.6–14.4]	14 [6.9–22.2]	< 0.0001	** < 0.0001**
SatO_2_ at admission, %	93 [90–96]	89 [83–93]	< 0.0001	** < 0.0001**
D dimer peak value, µg/L	4 [4–17.3]	4 [4–19.3]	0.782	> 1
TnI peak value, ng/L	29.5 [9.8–153]	123 [59–1070]	< 0.0001	** < 0.0001**
Presence of any comorbidity (*n*, %)	807/1245 (64.8%)	343/424 (80.9%)	< 0.0001	** < 0.0001**
Hypertension (*n*, %)	661/1240 (53.3%)	276/418 (66%)	< 0.0001	** < 0.0001**
Heart diseases (*n*, %)	135/1223 (11%)	93/410 (22.7%)	< 0.0001	** < 0.0001**
Previous PTCA (*n*, %)	68/967 (7%)	35/286 (12%)	< 0.0001	** < 0.0001**
Previous CABG (*n*, %)	29/965 (3%)	21/285 (7.4%)	< 0.0001	** < 0.0001**
Peripheral artery disease (*n*, %)	56/1240 (4.5%)	46/417 (11%)	< 0.0001	** < 0.0001**
Diabetes (*n*, %)	197/1240 (15.9%)	113/418 (27%)	< 0.0001	** < 0.0001**
Chronic obstructive pulmonary disease (*n*, %)	106/1240 (8.5%)	68/417 (16.3%)	< 0.0001	** < 0.0001**
Oncological history (*n*, %)	52/1239 (4.2%)	22/417 (5.3%)	0.356	> 1
Chronic renal failure (eGFR < 60)	54/959 (5.6%)	45/279 (16%)	< 0.0001	** < 0.0001**

Data are reported as median [Interquartile range] except otherwise specified

adj. *p* value: adjusted *p* value with Bonferroni’s correction

In boldface are adjusted *p* values

Participants were mostly men (1105/1669, 66.2%) with a median age of 67.5 [IQR, 58.5–77.4] years, with reduced median oxygen saturation (92% [IQR, 88–95%]). Hypertension was the most common comorbidity (937/1658, 56.5%).

**Fig. 1:**
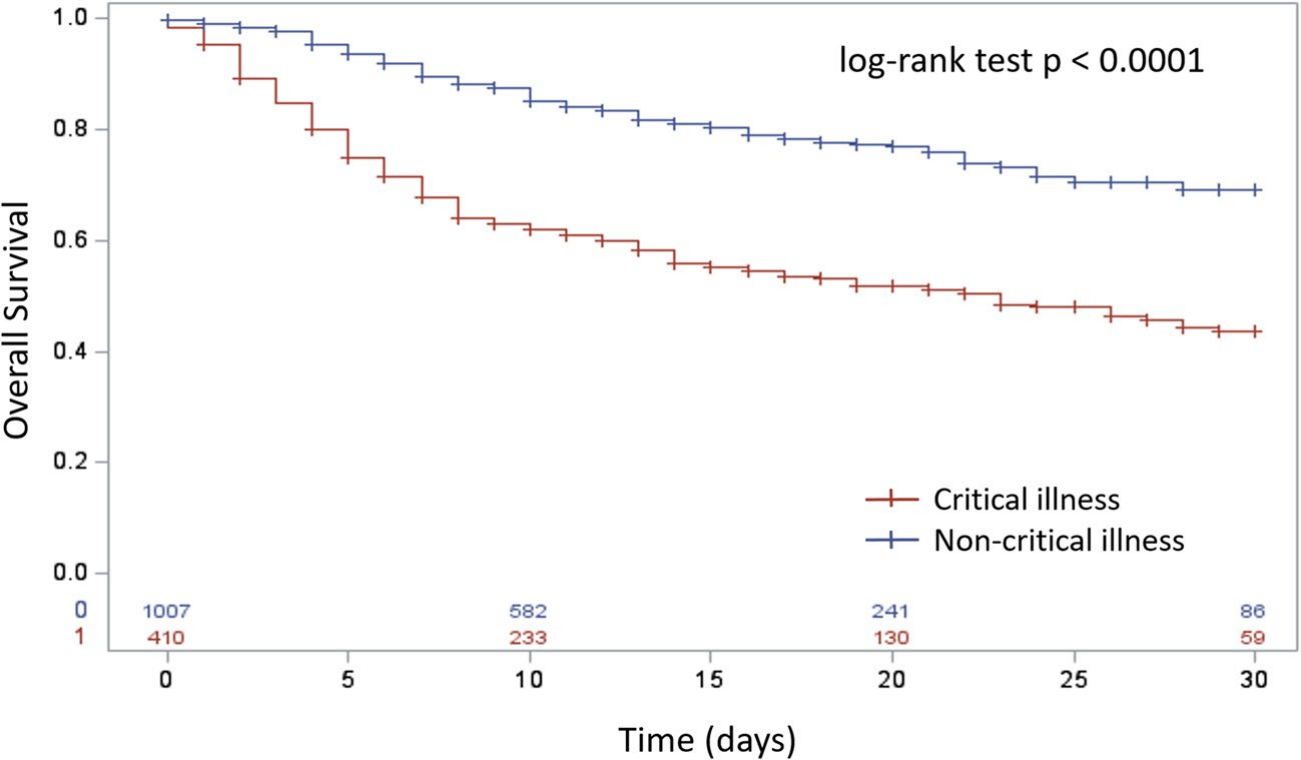
Overall survival in COVID-19 patients according to the severity of disease

Seventy-three patients (4.4%) were not hospitalized, 161 (9.6%) were admitted without the need for oxygen therapy, 543 (32.5%) were admitted with the need for oxygen therapy, 269 (16.1%) needed non-invasive ventilation (NIV), and 199 (11.9%) needed invasive ventilation. Median hospitalization length was 14 [IQR, 8–23] days. The mortality rate at 30 days was 24% (395/1669) in the overall population, significantly different according to the severity of disease (*p* = 0.0001) with a mortality rate of 17% in non-critically ill patients and 39% in critically ill patients (Fig. 1) assuming pneumonia involvement superior to 50% of lung volume as a surrogate marker for critical illness.

Non-survivors were significantly older (75 vs 65 years, adj. *p* value < 0.0001) and had worse inflammatory status with higher WBC (7350 vs 6590 WBC/mm^3^, adj. *p* value < 0.0001), LDH (450 vs 328 uL, adj. *p* value < 0.0001), CRP values (14 vs 7.6 mg/dL, adj. *p* value < 0.0001), higher prevalence of comorbidities (80.9%, vs 64.8%, adj. *p* value < 0.0001) and lower SatO2 levels at admission (89% vs 93%; adj. *p* value < 0.0001).

### Multiparametric CT characterization of high-risk patients

Patients suffered from pneumonia mainly involving less than 50% of lung volume (1337/1669, 67.1%). The prevalence of coronary atherosclerosis was high (1132/1669, 68%) with calcium score values higher than > 10 AU. Severe-to-moderate steatosis had a prevalence of 27.9% (465/1664), while HU values indicative of sarcopenia and osteoporosis were found in 35% (582/1661) patients and 55.4% (910/1642) patients, respectively (Table 1).

At multiparametric chest CT evaluation, non-survivors in comparison to survivors showed more severe pneumonia with an involvement higher than 50% in 50.9% vs 25.4% patients (adj. *p* value < 0.0001), higher coronary calcium score (131.9 [IQR, 5.3–581.2] AU vs 11.2 [IQR, 0–150.3] AU, adj. *p* value < 0.0001), higher prevalence of myosteatosis (52.1% vs 29.2%; adj. *p* value < 0.0001), and of osteoporosis (72.5% vs 49.5%; adj. *p* value < 0.0001) (Table 3).

**Table 3:** CT biomarkers of lung, cardiovascular, and metabolic features in Discharged and Deceased Patients

	Discharged **(***n*** = **1245)	Deceased (*n*** = **424)	*p* value	Adjusted *p* valued
Pneumonia score	2 [1-3]	3 [2, 3]	< 0.0001	** < 0.0001**
Patients with pneumonia > 50% (*n*, %)	316/1245 (25.4%)	216/424 (50.9%)	< 0.0001	** < 0.0001**
Agatston score	11.2 [0–150.3]	131.9 [5.3–581.2]	< 0.0001	** < 0.0001**
Agatston score 0 < 10 (*n*, %)	570/1168 (48.8%)	104/372 (28.0%)	< 0.0001	** < 0.0001**
Agatston score ≥ 10 < 1000 (*n*, %)	516/1168 (44.2%)	212/372 (57.0%)	< 0.0001	** < 0.0001**
Agatston score ≥ 1000 (*n*, %)	82/1168 (7.0%)	56/372 (15.1%)	< 0.0001	** < 0.0001**
Agatston score ≥ 1000, PCI and/or CABG (*n*, %)	157/1243 (12.6%)	108/424 (25.5%)	< 0.0001	** < 0.0001**
Paravertebral muscle attenuatation at D12 level, HU	41.8 [33.3–48.2]	35 [26.2–43.2]	< 0.0001	** < 0.0001**
Patients with myosteatosis (*n*, %)	362/1239 (29.2%)	220/422 (52.1%)	< 0.0001	** < 0.0001**
Liver attenuation, HU	47.8 [39.1–54.2]	46.3 [36.2–52.6]	0.004	**0.024**
Patients with hepatic steatosis (*n*, %)	330/1241(26.6%)	135/423 (31.9%)	0.035	0,21
D12 vertebral bone attenuation, HU	136.4 [99.9–171.7]	109 [76.2–138.3]	< 0.0001	** < 0.0001**
Patients with osteoporosis (*n*, %)	604/1220 (49.5%)	306/422 (72.5%)	< 0.0001	** < 0.0001**

Data are reported as median [Interquartile range] except otherwise specified

adj. *p* value: adjusted *p* value with Bonferroni’s correction

In boldface are adjusted *p* values

**Fig. 2:**
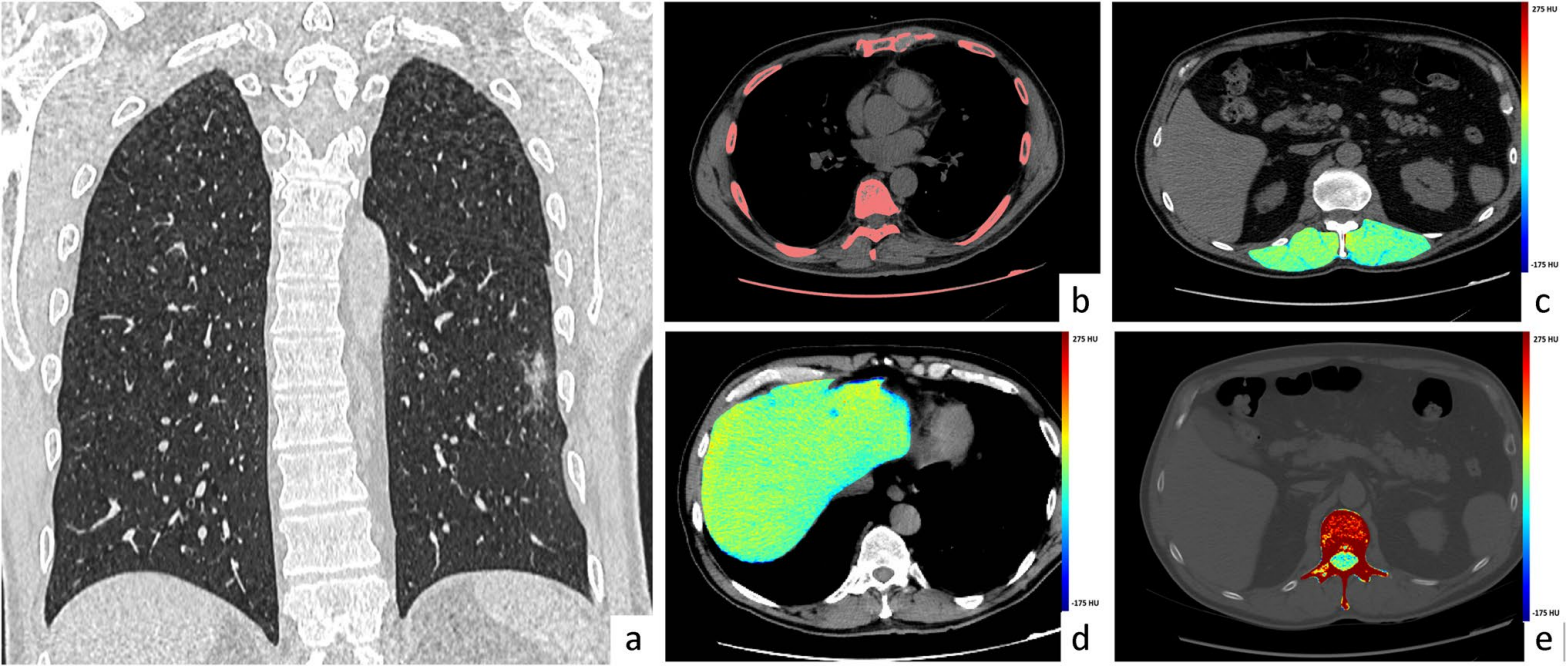
Exemplifying patients treated without the need for oxygen therapy. Male patient, 56 y.o. Vital signs were stable at the moment of admission to the hospital, and SaO2 in ambient air was 95%. C-Reactive Protein was 1.15 mg/dL. CT scan was performed two days after admission and showed a minimal lung parenchymal involvement with few patchy areas of ground glass opacity (Pneumonia score 1, **a**); moreover, the CT scan revealed absent coronary calcium (Agatston score, **b**), along with the absence of myosteatosis (**c**), liver steatosis (**d**), and osteoporosis (**e**), based on mean tissues attenuation enhanced by colorimetric maps of HU documenting HU in range of normality for paravertebral muscles (48 HU, **c**), liver (51 HU, **d**) and trabecular bone (181 HU, **e**). The patient was admitted to the hospital and discharged after three days without worsening of clinical condition and without the need for oxygen therapy

**Fig. 3:**
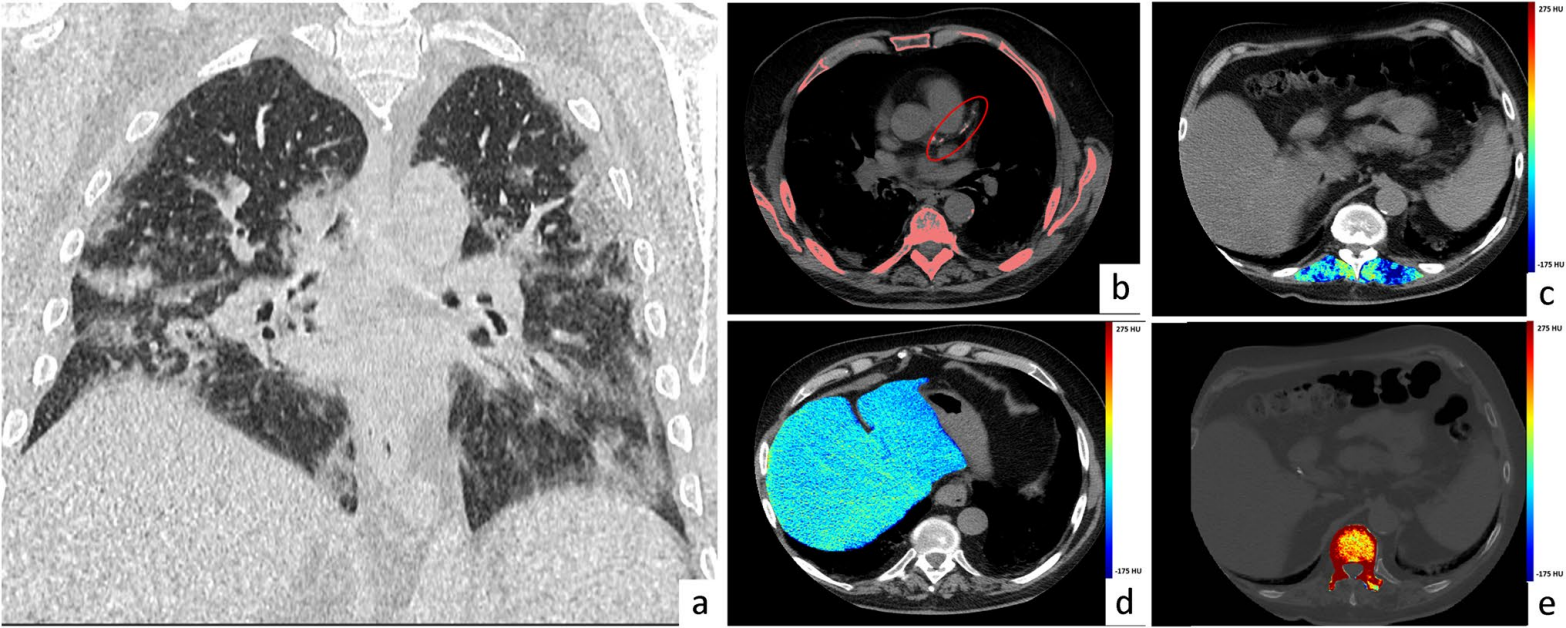
Exemplifying patient treated with non-invasive ventilation. Male patient,75 y.o., with a clinical history of hypertension and diabetes. At admission to the hospital SaO2 in ambient air was 80%. Main laboratory data were: WBC 6200/mm3, C-Reactive Protein 2.64 mg/dL. CT scan was performed after two days after admission, revealing a moderate involvement of the lung parenchyma (Pneumonia score 3, **a**), a low coronary artery calcium score (Agatston score 46, **b**), reduced liver attenuation, indicating the presence of steatosis (mean density of right and left lobe 25.5 HU, **d**), presence of myosteatosis (mean attenuation of paravertebral muscles 23 HU, **c**), and osteoporosis (bone density 126 HU, **e**). The patient was admitted to the hospital, and during the hospitalization needed non-invasive ventilation. He was discharged after 34 days

**Fig. 4:**
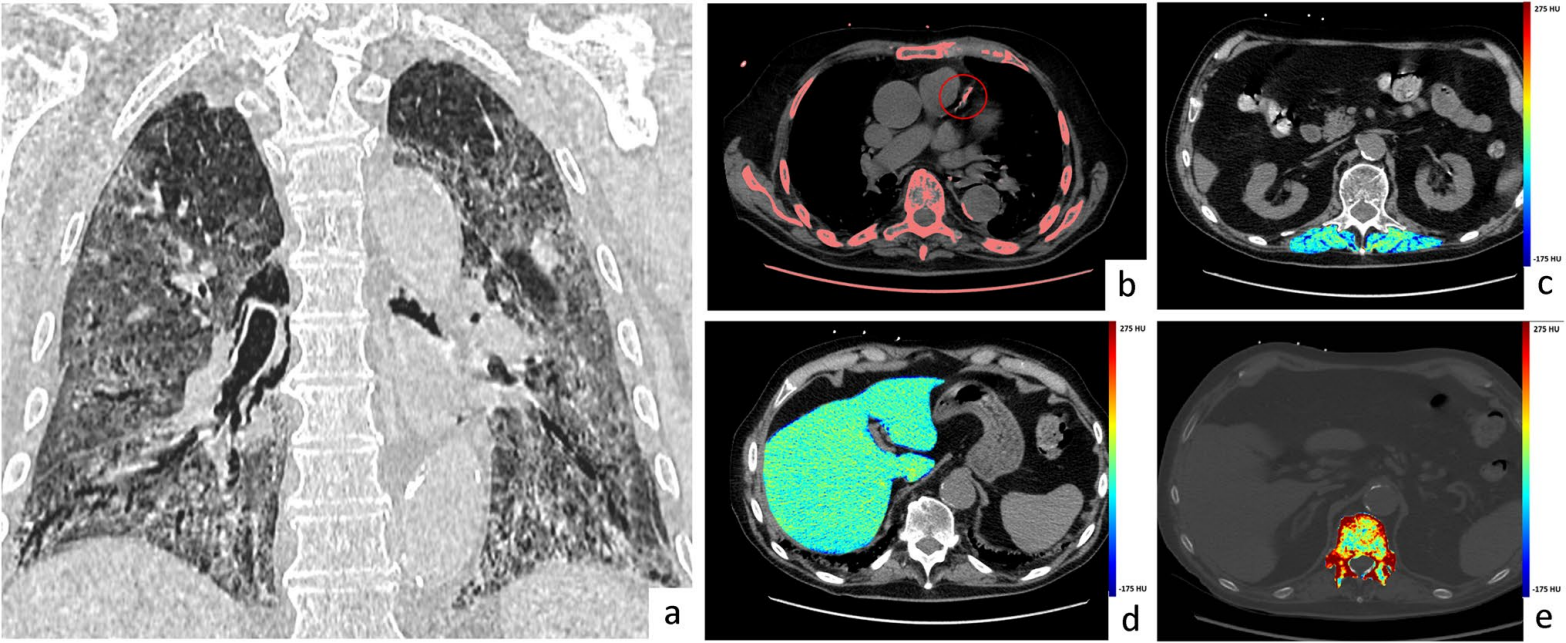
Exemplifying case of admitting chest CT findings in non-survivor. Male patient, 84 y.o., with a clinical history of hypertension, diabetes, and chronic kidney disease. At the hospital admission, vital signs were stable but SaO2 in ambient air was 87%. Laboratory data were collected, in particular: WBC 13,000/mm^3^, C-reactive protein 1.18 mg/dL. CT scan was performed the same day of admission, revealing an important involvement of the lung parenchyma (Pneumonia score 4, **a**) along with a moderate coronary artery calcium score (Agatston score 207, **b**), a reduced attenuation of paravertebral muscles, indicating the presence of myosteatosis (mean attenuation 27.5 HU,** c**), a liver attenuation suggestive for steatosis (mean density of right and left lobe 40 HU, **d**), indicating the absence of steatosis, and reduced trabecular attenuation of D12 vertebral bone (94 HU, **e**). The patient was admitted to Intensive Care Unit and after 27 days of hospitalization died

Severity of pneumonia extension and of cardiovascular risk (calcium score > 1000 AU and previous revascularization), myosteatosis, fatty liver, and osteoporosis were also associated with different kind oxygen therapy (no oxygen therapy, oxygen therapy, NIV, and orotracheal intubation) during the hospitalization, as shown in Table 4 (Figs. 2, 3, and 4).

**Table 4:** Radiological characteristics of patients according to different treatments and outcomes

	Non hospitalized **(***n*** = **73)	No oxygen therapy **(***n* **= **161**)**	Oxygen therapy **(***n*** = **543)	Non Invasive Ventilation **(***n* **= **269)	Invasive Ventilation **(***n*** = **199)	Death **(***n*** = **424)	*p* value
Age (mean ± Sd)	56.42 ± 14.76	63.04 ± 14.44	67.66 ± 12.95	65.21 ± 11.26	61.18 ± 11.30	75.39 ± 10.21	< 0.001
Male sex (*n*, %)	30 (41.1%)	61 (37.9%)	234 (43.1%)	71 (26.4%)	47 (23.6%)	108 (25.5%)	< 0.001
Pneumonia score	1 [1, 2]	1 [1, 2]	2 [1, 2]	2 [2, 3]	3 [2–4]	3 [2, 3]	< 0.001
Pneumonia score ≥ 3 (*n*, %)	6 (8.2%)	12 (7.5%)	75 (13.8%)	92 (34.2%)	131 (65.8%)	216 (50.9%)	< 0.001
Agatston score	0 [0–23]	9,8 [0–130.7]	19,6 [0–258]	12,8 [0–140.8]	5,5 [0–100.6]	131,7 [5.2–579.2]	< 0.001
Agatston score 0 < 10 (*n*, %)	48 (69.6%)	76 (50.3%)	219 (43.1%)	123 (48.4%)	105 (55.9%)	104 (28.0%)	< 0.001
Agatston score ≥ 10 < 1000 (*n*, %)	16 (23.2%)	67 (44.4%)	247 (48.6%)	112 (44.1%)	75 (39.9%)	212 (57.0%)	< 0.001
Agatston score ≥ 1000 (*n*, %)	5 (7.2%)	8 (5.3%)	42 (8.3%)	19 (7.5%)	8 (4.3%)	56 (15.1%)	< 0.001
Agatston score ≥ 1000, PCI and/or CABG (*n*, %)	9 (12.3%)	18 (11.2%)	77 (14.2%)	34 (12.6%)	19 (9.5%)	108 (25.5%)	< 0.001
Liver attenuation, HU	53 [44 –60]	50.4 [44–56.8]	47.2 [38.4–53]	46,5 [33.2–53.5]	46.8 [38.8–53.7]	46.3 [36.2–52.6]	< 0.001
Hepatic steatosis (*n*, %)	9 (12.3%)	27 (14.4%)	153 (28.2%)	85 (31.7%)	56 (28.1%)	135 (31.9%)	< 0.001
D12 vertebral bone attenuation, HU	167.3 [128–198]	133 [101–173]	125 [91–161]	138 [99–171]	146 [119–186]	109 [76–138]	< 0.001
Osteoporosis (*n*, %)	19 (28.8%)	80 (51.3%)	308 (57.32%)	126 (47.9%)	71 (36%)	306 (72.5%)	< 0.001
Paravetrebral muscle attenuation at D12 level, HU	46 [37–52]	43 [33–50]	40 [31–46]	43,5 [35–50]	42 [36–48]	35 [26–43]	< 0.001
Myosteatosis (*n*, %)	13 (18.1%)	44 (27.8%)	184 (33.9%)	76 (28.5%)	51 (25.6%)	220 (52.1%)	< 0.001

In detail, at post hoc analysis pneumonia severity was not significantly different in hospitalized and non-hospitalized patients without the need for oxygen therapy (*p* = 1), but differed according to the kind of oxygen therapy, with increased severity associated with increased aggressiveness of treatment (always *p* < 0.001). Similarly, non-survivors had slight but significantly more extensive pneumonia than survivors submitted to orotracheal intubation (*p* = 0.023). Non-survivors showed higher cardiovascular risk (calcium score > 1000 AU and previous revascularization) than survivors independently by the treatment (always *p* < 0.001). Patients’ candidate for oxygen therapy and NIV had a higher rate of myosteatosis compared to patients not needing oxygen supply (*p* < 0.001 and *p* = 0.043, respectively). Moreover, non-survivors had more frequent myosteatosis than survivors candidate for NIV (*p* < 0.001) and orotracheal intubation (*p* < 0.001).

Non-hospitalized patients had a lower prevalence of liver steatosis than hospitalized patients and non-survivors (always *p* ≤ 0.001) as well as of osteoporosis (always *p* < 0.05).

### Outcome prediction

At multivariable binary logistic regression analysis including only clinical data, independent predictors of unfavourable outcome were: male sex, older age, CRP, and SaO2 with an AUC of 0.800 (95% CI 0.774–0.825; *p* < 0.0001). At multivariable binary logistic regression analysis including clinical and CT parameters, independent predictors of outcome were male sex, older age, CRP, SaO2 together with pneumonia involvement > 50%, liver steatosis, and osteoporosis with AUC of 0.815 (95% CI 0.790–0.839; *p* < 0.0001) Table 5. This model showed significantly higher AUC than the model including only clinical variables (*p* = 0.001). It has been tested in patients with and without critical illness, and it was confirmed superior to the model including only clinical variables in patients with non-critical illness (AUC 0.801 vs 0.788; *p* = 0.0198), while it showed similar performances in patients with a critical illness (AUC 0.802 vs 0.795, *p* = 0.2338).

**Table 5:** Multivariable binary logistic regression to build a clinical model and clinical CT model

Variable	OR	95% CI	*p* value
Clinical model			
Age	1.087	1.073–1.102	< 0.0001
Male sex	1.891	1.402–2.550	< 0.0001
CRP	1.005	1.002–1.008	0.002
SaO_2_	0.908	0.888–0.928	< 0.0001
Clinical-CT model			
Male sex	1.872	1.375–2.550	< 0.0001
Age	1.085	1.069–1.101	< 0.0001
CRP	1.005	1.002–1.008	0.003
SaO_2_	0.932	0.911–0.954	< 0.0001
Pneumonia	2.519	1.865–3.402	< 0.0001
Myosteatosis	1.244	0.937–1.653	0.132
Steatosis	1.382	1.027–1.859	0.033
Osteoporosis	1.519	1.103–2.093	0.010
Agatston score ≥ 1000, PCI and/or CABG	1.330	0.947–1.868	0.100

## Discussion

Cardiovascular and metabolic comorbidities have been demonstrated to affect the outcome of COVID-19 patients [15, 16, 23, 24, 26]. However, a detailed collection of patient comorbidities could be challenging in an emergency for overwhelmed physicians, for several issues related to the patient clinical condition, and for the potential risk of subclinical comorbidities previously unknown.

CT offers the possibility to non-invasively identify cardio-metabolic risk factors based on disease-related organ attenuation modifications [15, 16, 23, 24, 26], that could be used to improve patients’ risk stratification.

Therefore, from chest CT routinely performed for the evaluation of COVID-19 pneumonia, a wide spectrum of information about patient health status could be potentially extracted and used to improve the non-invasive phenotypization of high-risk patients.

At this aim, we analysed non-contrast chest CT studies of 1669 consecutive COVID-19 patients performed within 72 h from hospital arrival with the extraction of CT biomarkers of cardiovascular risk and metabolic status and they resulted in being associated with patients’ prognosis (discharge vs death).

In particular, non-survivors, besides more severe pneumonia, had higher coronary artery calcium burden and higher prevalence of liver steatosis, myosteatosis, and osteoporosis. These CT biomarkers resulted associated with the severity of oxygen treatment during hospitalization and in-hospital death. The integration of these imaging biomarkers with clinical predictors of outcome in a single multivariable model showed higher diagnostic performance compared to a model including only clinical variables in the entire population (AUCs 0.815 vs 0.800; *p* < 0.0001), and in the subgroup of non-critically ill patients (AUCs 0.801 vs 0.788; *p* = 0.0198).

All the imaging biomarkers included in the present study measure changes in tissue composition associated with lifestyle-related and aging-related conditions, including cardiovascular diseases, diabetes, osteoporosis, obesity, and fragility, which are clinical conditions associated with greater risk of COVID-19 infection and more severe symptoms [27, 28].

The calcium score is a well-established CT biomarker capable of identifying individuals at higher risk of cardiovascular events [29] by providing evidence of coronary artery disease also if previously clinically unknown [8, 10]It can be measured from non-ECG gated chest CT with a manual or semiautomatic approach [12, 15] or using machine learning algorithm [30]. Calcium score resulted in more severe illness in COVID-19 [8, 10, 12] and a higher rate of in-hospital mortality, myocardial infarction, and cerebrovascular event during hospitalization[10, 12] with superior risk stratification performance compared to a clinical cardiovascular risk assessment [8, 10] as also supported by a recent meta-analysis on 3769 patients, calcium scoring can help in stratifying COVID-19 patients, allowing earlier interventions in rapidly developing illnesses[31].

Increased cardiovascular risk is often associated with dysmetabolism and liver steatosis. A previous study found 40 HU as the most accurate CT cut-off value for moderate-to-severe macro-vesicular steatosis [23] and it represents an objective and non-invasive mean for detecting asymptomatic hepatic steatosis, whereas clinical risk factor assessment is unreliable [23, 26]. This method would lead to the underestimation of the overall prevalence of fatty steatosis in our population for missing cases of mild fatty liver [32] if compared to other threshold values as < 51 HU or other parameters as liver/spleen ratio < 1.0 [32]. However, the threshold used was found to reflect 30% of liver fat content [32] and to depict patients at higher risk of disease progression [23, 26, 33].

Non-alcoholic fatty liver disease (NAFLD)[34] is often associated with metabolic dysregulation, including obesity, diabetes, dyslipidaemia, and insulin resistance. NAFLD is considered a risk factor for developing SARS-CoV-2 infection and for disease progression, being associated with a higher rate of severe disease, hospitalization, and death [35-37].

During hospitalization, elevation in transaminase values has been reported in patients with COVID-19 in response to direct liver damage and drugs’ cytotoxic effect [38] with an overall incidence of liver damage from 14.8 to 53%, resulting more frequent in severe than in mild disease [39]. In patients with NAFLD, the rate of liver dysfunction is higher than in patients without (70% versus 11.1%) [35], and it is attributed to NAFLD-related impaired liver function [40]. In a series of 14 autopsies [37], liver steatosis was observed in almost all deceased COVID-19 patients (12/14). Liver steatosis was also highly prevalent after hospital discharge, with potential long-term metabolic and cardiovascular health implications in long COVID-19 disease [41].

Additionally, in our cohort, higher prevalence of myosteatosis and osteoporosis was observed in patients with more severe oxygen treatment and in non-survivors. Myosteatosis and osteoporosis are age-associated declines in muscle mass, strength, quality, and bone density, hence are more likely to occur in older populations [27]; however, their aetiology is multifactorial and they could be associated with several pathologies including type 2 diabetes mellitus and cardiovascular disease, where inappropriate nutrition and sedentary lifestyle play a central role [27].

Myosteatosis is associated with worse prognosis in several conditions such as major surgery and oncological and cardiovascular disease and is documented in some previous studies on COVID-19; it is a negative prognostic factor for severe COVID-19, associated with in-hospital mortality[42].

Moreover, in non-COVID-19 settings, myosteatosis was associated with a higher risk of respiratory failure and worse outcomes in mechanically ventilated patients [43] probably for the potential impact on respiratory muscles.

Myosteatosis measured by muscle mean attenuation which is lower when fat content is higher [17]. was also associated with a higher rate of post-acute sequelae in COVID-19 patients. In particular, myosteatosis, rather than muscle mass, was associated with functional impairment at 3 [44] and 6 months [45] after hospital discharge.

In order to provide a more comprehensive morphometric assessment of patients, the estimation of myosteatosis could be combined with the estimation of visceral and subcutaneous fat from CT, which has been demonstrated to improve non-invasive risk stratification in other settings such as oncology and surgery [46], especially when derived by abdominal CT, for better stratification of cardiometabolic risk [47]. Moreover, a recent study on COVID-19, found also that fat attenuation is associated to serological markers of systemic inflammation and to more severe disease [11].

Despite the potential clinical utility of the extraction of all these CT opportunistic biomarkers for cardiometabolic risk stratification, the clinical application is still limited because of manual segmentation, which is time-consuming and affected by a certain degree of subjectivity. Therefore, the development of AI tools for the simultaneous automatic extraction of these biomarkers as already developed for some parameters such as lung, coronary calcium [30], fatty liver [48], and for abdominal CT [47], it would potentially improve data reliability and clinical applicability and its usage in large-scale population-based screening[47].

Our study has some limitations. First is the retrospective nature of the study and the lack of validation. However, these data were collected during the first Italian pandemic wave, when disease prevalence and severity were higher and no potentially confounding factors such as an effective treatment were available. Moreover, CT parameters were not compared to disease-specific laboratory or functional testing, because of challenges in data collection during the emergency and due to limited standardization of clinical approaches and testing among the hospitals involved. However, the robustness of our results is supported by the use of previously validated cut-off values, which allowed to overcome the absence of detailed clinical data and specific laboratory tests with the additional advantage of objectively identifying subclinical and previously unknown conditions. Additionally, we think that this approach could be useful in risk stratification of patients suffering from diseases affecting both the respiratory and cardiovascular systems such as cardiogenic pulmonary edema.

In conclusion, chest CT performed for the assessment of lung parenchyma involvement in COVID-19 can provide a comprehensive phenotypization of patient comorbidities and risk profile, with better stratification of risk compared to the sole evaluation of pneumonia. Further studies are needed to determine the capability of this multiparametric approach to predict long-term COVID-19 sequelae.

## Declarations

### Guarantor

The scientific guarantor of this publication is Antonio Esposito.

### Statistics and biometry

Simone Barbieri has significant statistical expertise.

### Study subjects or cohorts overlap

Some study subjects or cohorts have been previously reported in “Esposito A, Palmisano A, Toselli M, et al. Chest CT-derived pulmonary artery enlargement at the admission predicts overall survival in COVID-19 patients: insight from 1461 consecutive patients in Italy. *Eur Radiol.* 2021 Jun;31(6):4031–4041. https://doi.org/10.1007/s00330-020-07622-x.”; “Giannini F, Toselli M, Palmisano A, et al. Coronary and total thoracic calcium scores predict mortality and provides pathophysiologic insights in COVID-19 patients. *J Cardiovasc Comput Tomogr.* 2021 Sep-Oct;15(5):421–430. https://doi.org/10.1016/j.jcct.2021.03.003”; and “Palmisano A, Vignale D, Boccia E. AI-SCoRE (artificial intelligence-SARS CoV2 risk evaluation): a fast, objective and fully automated platform to predict the outcome in COVID-19 patients. *Radiol Med.* 2022 Aug 29:1–13. https://doi.org/10.1007/s11547-022-01518-0.”;

### Methodology

• retrospective

• observational

• multi-center study
